# Decreased Neuromuscular Function and Muscle Quality along with Increased Systemic Inflammation and Muscle Proteolysis Occurring in the Presence of Decreased Estradiol and Protein Intake in Early to Intermediate Post-Menopausal Women

**DOI:** 10.3390/nu16020197

**Published:** 2024-01-07

**Authors:** Darryn S. Willoughby, Christine Florez, Jaci Davis, Nikolas Keratsopoulos, Morgan Bisher, Mandy Parra, Lemuel Taylor

**Affiliations:** 1 School of Health Professions, University of Mary Hardin-Baylor, Belton, TX 76513, USA; 2 School of Exercise and Sport Science, University of Mary Hardin-Baylor, Belton, TX 76513, USA; christine.florez@ttu.edu (C.F.);

**Keywords:** muscle strength, muscle mass, motor unit, neuromuscular junction, aging

## Abstract

Menopause causes a reduction in estradiol (E2) and may be associated with neuromuscular degeneration. Compared to pre-menopausal (PRE-M) women, this study sought to determine dietary protein intake and whether lower levels of circulating E2 in post-menopausal women (POST-M) were occurring alongside increased levels of biomarkers of axonal and neuromuscular junction degeneration (NMJ), inflammation, muscle protein degradation, and reduced indices of muscle quality and performance. Employing a cross-sectional design, PRE-M (*n* = 6) and POST-M (*n* = 6) dietary analysis data were collected and participants then donated a blood and urine sample followed by assessments for body composition, motor unit activation, and muscle performance. Independent group *t*-tests were performed to determine differences between groups (*p* ≤ 0.05). In POST-M women, E2, motor unit activity, muscle quality, and muscle performance were significantly less than those for PRE-M women; however, the levels of *c*-terminal fragment of agrin, tumor necrosis factor-α, and urinary titin were significantly greater (*p* < 0.05). POST-M women were also shown to be ingesting fewer total calories and less protein than PRE-M (*p* < 0.05). Reduced E2 and dietary protein intake in POST-M women occurs in conjunction with increased levels of biomarkers of NMJ degradation, inflammation, and muscle proteolysis, which may be associated with reduced motor unit activation and muscle quality.

## 1. Introduction

Menopause occurs due to a decrease in the endogenous synthesis of the estrogenic hormone, 17-β estradiol (E2) [1]. This senescence is due to a reduction in the activity of the hypothalamo–pituitary–gonadal (HPG) axis. The role of E2 in the activity and maintenance of the uterine cycle is well known, but it also plays a critical role in preventing sarcopenia and maintaining neuromuscular function through its antioxidant properties and by serving to protect the clustering of acetylcholine (Ach) receptors at the neuromuscular junction (NMJ) [2]. Because of the antioxidant properties of E2 [3], the reduction in endogenous E2 associated with menopause leads to oxidative stress and a subsequent increase in the expression of the pro-inflammatory mediators, tumor necrosis factor-α (TNF-α) [4] and nuclear factor kappa кB (NF-кB) [5], which can detrimentally impact muscle mass and function [6]. There is evidence demonstrating that a reduction in E2 triggers the release of TNF-α, suggesting that the decline in E2 associated with menopause can mediate inflammatory-regulated sarcopenia, thereby negatively impacting muscle quality (MQ) and muscular function [7].

Sarcopenia results in a progressive decline in MQ, mass, and performance (strength, power, endurance) that occurs during aging [8]. It has also been suggested that an inadequate protein intake might be associated with sarcopenia in older adults [9]. This deterioration in muscle function accompanying sarcopenia has been shown to occur due to a decrease in motor unit activation and a reduction in the number of functioning motor units [10]. Furthermore, these declinations occur at an exponentially faster rate at the onset of menopause once E2 levels are dramatically decreased [11] and are attributed, in part, to a reduction in Ach receptor clustering and a subsequent degeneration of the NMJ. This detrimental impact on the NMJ leads to an overall decline in motor unit and muscle fiber activation and decrements in muscle performance [12]. Late post-menopause (>10 years of amenorrhea) is more associated with pronounced sarcopenia; however, impacts on neuromuscular degradation during early to intermediate post-menopause (<10 years of amenorrhea) are not well known.

Within α-motor neurons, agrin and neurofilament light chain (NfL) are proteins playing important roles in the integrity of the NMJ and axon, respectively. Agrin is a proteoglycan expressed by motor neurons. It is transported down the axon released from the pre-synaptic terminal into the synaptic cleft, where it becomes an essential component of the basal lamina of the post-synaptic terminal, and is involved in initiating Ach receptor clustering [13]. The degradation of agrin yields a *C*-terminal agrin fragment (CAF), produced when agrin is cleaved by the trypsin-like serine protease neurotrypsin (NT), an enzyme that has been shown to be up-regulated by TNF-α during situations of inflammation and oxidative stress [14,15]. Circulating CAF is a biomarker for NMJ degradation and subsequent muscle weakness and atrophy in humans [16,17].

NfL is a type of neurofilament found in the neuroplasm and is primarily a structural protein of myelin that surrounds axons. The elevation of NfL in the circulation or cerebrospinal fluid is associated with axonal injury and is considered as a biomarker of neurological/axonal (but not NMJ) degeneration [18]. Studies have shown higher concentrations of NfL in populations with neurological diseases, with one study observing elevated levels of NfL in sarcopenic populations as compared to healthy populations [19].

Titin is a structural protein which assists in maintaining architectural integrity of the sarcomere by anchoring the interconnection of the actin-containing thin filaments and myosin-containing thick filaments to the Z-line. During muscle proteolysis, titin is degraded into a smaller 25 kDa fragment to form titin *N*-terminal fragment (TNTF) which is excreted in the urine and is an effective biomarker used to assess muscle proteolytic degradation and atrophy in clinical situations such as interstitial lung disease [20], non-alcoholic fatty liver disease [21], and post-intensive care syndrome [22].

Compared to pre-menopausal (PRE-M) women, the purposes of this study were two-fold, the first being to determine whether lower levels of circulating E2 in post-menopausal (POST-M) occurred with increased levels of biomarkers of axonal and NMJ degeneration (NfL, CAF and NT), pro-inflammatory status (TNF-α), and muscle protein degradation (UTNF). In addition, we sought to determine whether a reduced E2 level in POST-M women also occurred with reductions in muscle mass, MQ, motor unit/muscle activation, and muscle performance.

## 2. Methods

### 2.1. Experimental Design

Employing a two-group cross-sectional design, participants deemed eligible for the study following their review of exclusionary criteria were required to visit the lab for one testing session where hemodynamic (heart rate and blood pressure) and anthropometric (height, body mass, fat mass, and fat-free mass) measurements and dietary analysis data were collected and recorded upon arrival by study personnel. Participants then donated a non-fasted blood and urine sample between 1 and 3 pm followed by assessments for body composition, electromyography (EMG), and muscle strength and endurance.

### 2.2. Participants

Twelve apparently healthy, untrained women who verbally categorized themselves as either pre- or post-menopausal (no menstrual activity for 12 consecutive months prior to the study) volunteered to serve as study participants; all 12 participants completed the study. Participants were divided into one of two groups: a pre-menopausal group (PRE-M (*n* = 6; age: 23.33 ± 4.32 y; height: 160.72 ± 1.07 cm; total body mass [TBM]: 60.53 ± 11.57 kg)) and a post-menopausal group (POST-M (*n* = 6; age: 61 ± 3.32 y; height: 164.34 ± 0.18 cm; TBM: 77.12 ± 9.31 kg, post-menopausal: 9.34 ± 9.56 y)). The main inclusion criteria required participants to not be receiving hormone replacement therapy for POST-M women or any form of pharmacological contraceptive for PRE-M women, and to have not participated in structured resistance training in the 12 months immediately prior to the study commencing. Each participant read and signed an informed consent document. Additionally, participants were screened for any medical conditions that would exclude them from participation (such as current smoker; known pregnancy (for pre-menopausal); currently taking any form of oral contraceptive (for pre-menopausal); currently taking any form of hormone replacement (for post-menopausal); had been involved in a habitual exercise training program which utilized the lower and upper body within the previous year; had orthopedic limitations that would limit participation in exercise; had any known metabolic disorder including heart disease, arrhythmias, diabetes, thyroid disease, or hypogonadism; had previously suffered from a cardiovascular event such as a heart attack or stroke; had a bleeding disorder, history of pulmonary disease, hypertension, hepatorenal disease, musculoskeletal disorders, neuromuscular/neurological diseases, autoimmune disease, cancer, peptic ulcers, anemia, or chronic infection (e.g., HIV); or were taking any blood thinning (e.g., warfarin, Jantoven, etc.), heart, pulmonary, thyroid, anti-hypertensive, endocrinologic (e.g., thyroid, insulin, etc.), emotional/psychotropic (e.g., Prednisone, Ritalin, Adderall), or neuromuscular/neurological medication) by completing a health history questionnaire and a physical activity readiness questionnaire (PAR-Q). Only participants considered as low risk for cardiovascular disease and with no contraindications to exercise as outlined by the American College of Sports Medicine (ACSM) and had not consumed any type of nutritional supplements (excluding multi-vitamins) one month prior to the study could participate. Approval to conduct the study was granted on 15 April 2022 by the Institutional Review Board for the Protection of Human Subjects in Research (IRB #204). All experimental procedures conformed to policies according to the World Medical Association’s Declaration of Helsinki.

### 2.3. Venous Blood Collection

Venous blood samples were obtained from the antecubital vein into a 10 mL collection tube using a serum separator tube using a standard vacutainer apparatus. Blood samples were then centrifuged (Clinical 100, VWR, Atlanta, GA, USA) for 15 min at 1230× *g* after sitting at room temperature for 20 min. Using a transfer pipette, serum was aliquoted into separate microfuge tubes (Thermo Fisher Scientific, Austin, TX, USA) and frozen at −80 °C for later analysis. For PRE-M women, blood samples were collected during the follicular phase of their uterine cycle where E2 levels during this point of the cycle are typically ~350 pg/mL.

### 2.4. Urine Collection

Participants were instructed to collect a mid-stream urine sample in a provided sterile urine collection cup (Cole Parmer, Vernon Hills, IL, USA). The urine samples were extracted from the cups using a transfer pipette, then placed into a separate microfuge tube and frozen at −80 °C for later analysis.

### 2.5. Dietary Analysis

Participants were required to provide a consecutive three-day dietary recall in an effort to determine the average daily kilocalories and macronutrient consumption of carbohydrate, fat, and protein in the diet at the time of the study. Participants were instructed not to make any changes to their typical diet during the course of the three days. Based on approximated serving sizes, food logs were evaluated by study personnel consisting of a certified exercise/sport nutritionist (certified through the International Society of Sports Nutrition, www.sportsnutritionsociety.org, St. Petersburg, FL, USA) and estimations of caloric intake performed using MyFitnessPal v23.25.0 (MyFitnessPal, Inc., Austin, TX, USA).

### 2.6. Muscle Strength and Endurance

Protocols to establish one repetition maximums (1RM) for upper- and lower-body strength were conducted in accordance with guidelines set forth by the National Strength and Condition Association (NSCA). Following a five-minute stationary treadmill warm-up, participants performed ten repetitions at 50% of their estimated 1RM on a seated leg press and seated chest press (Cybex, Franklin Park, IL, USA). After a one-minute rest, participants performed five repetitions at 70% of an estimated 1RM leg press/chest press. After three minutes of rest, participants began their 1RM leg press/chest press attempts. Following each successful lift, the load was increased 5–10%. If the lift was successful, the participant rested for two minutes before attempting the next weight increment. This procedure continued until the participant failed to complete a successful lift. The 1RM was then recorded as the maximum weight that the participant was able to lift for one repetition. Participants were given five attempts to determine the 1RM. After a five-minute recovery period, both exercises were then followed by the performance of repetitions to failure (RTF) at 70% of the leg press and chest press 1RM for the assessment of lower- and upper-body muscle endurance.

### 2.7. Electromyography

Using EMG (Biopac Systems Inc., Goleta, CA, USA), surface electrodes (Ag/AgCl) of 10 mm diameter were applied at the approximate muscle belly of the vastus lateralis (VL) and the rectus femoris (FR). The areas were shaved, if necessary, abraded, and cleaned with isopropyl alcohol prior to electrode placement. While sitting on a table with the knees in slight flexion and the upper body slightly bent backward, anatomical landmarks were palpated to determine the muscle belly’s approximate location. Specific locations on both muscles were then determined using measurement tape and indicated with marker pen. Electrodes were then placed in a bipolar configuration with the grounding/reference electrode located on the medial and lateral tibial epicondyles. The collection electrodes were placed in the approximate mid-belly of each muscle, and for the RF and VL, this was 1/2 (50%) and 2/3 (67%), respectively, in the direction of the distance from the anterior superior iliac spine to the superior patellar border [23]. Using an EMG analysis software module, version ACKEMG (Biopac Systems Inc., Goleta, CA, USA), activation patterns to determine motor unit recruitment were recorded, and then were normalized and filtered through a high band pass. Mean and peak activation values were then recorded for each muscle during 1RM strength testing and throughout the duration of the muscle endurance testing (RTF at 70% 1RM) session.

### 2.8. Body Composition Assessment

Total body mass (TBM) and height were assessed using a standard scale (Tanita TBF-310, Arlington Heights, IL, USA) and stadiometer (Seca 264, Hamburg, Germany), respectively. The percent body fat (PBF), fat mass (FM), fat-free mass (FFM), and visceral fat (VFM) were determined using DEXA (Horizon W, Hologic, Bedford, MA, USA). Quality control calibration procedures were performed on a spine phantom (Hologic X-CALIBER Model DPA/QDR-1 anthropometric spine phantom) and a density step calibration phantom prior to each testing session. Total body water was determined by means of bioelectric impedance analysis (Biospace 770, InBody USA, Cerritos, CA, USA) using a low-energy, high-frequency current (500 micro amps at a frequency of 50 kHz).

### 2.9. Muscle Quality

Muscle strength using 1RM values for both the upper body (using the chest press) and for the lower body (using leg press) was expressed relative to total body mass for assessments of upper- and lower-body relative strength. These values were then used to quantify muscle quality (MQ) of the upper and lower body. Defined as the amount of strength per unit of muscle mass, MQ is a novel index of muscle performance [24]. Since the chest press employs muscle groups from the upper arms, shoulders, and trunk, upper-body MQ was calculated using upper-body relative strength divided by the sum of right and left arm fat free mass (FFM) and trunk FFM (MQ_UPPER_ = RS_UPPER_/(FFM_LEFT ARM_ + FFM_RIGHT ARM_ + FFM_TRUNK_)) [25]. Lower-body MQ was calculated similarly, using lower-body relative strength values divided by the sum of left leg FFM and right leg FFM (MQ_LOWER_ = RS_LOWER_/(FFM_LEFT LEG_ + FFM_RIGHT LEG_)).

### 2.10. Serum and Urinary Proteins and Hormones

From the blood samples obtained, commercially available enzyme-linked immunoabsorbent assay (ELISA) kits (MyBiosource Inc., San Diego, CA, USA) were used to assess neurofilament light chain (NfL (cat# MBS9399603)), *C*-terminal agrin fragment (CAF (cat# MBS7606926)), estradiol (E2 (Cat# MBS580165)), neurotrypsin (NT (cat# MBS2504321)), and tumor necrosis factor-α (TNF-α (cat# MBS2502004)). From the urine samples obtained, a commercially available ELISA kit was used to assess titin (TNTF (Cat# MBS762344)) and a colorimetric assay kit (Cayman Chemical Col., Ann Arbor, MI, USA, Cat# 500701) was used to assess creatinine. The TNTF concentration was expressed relative to urinary creatinine to adjust for kidney function [22]. Each sample was analyzed in duplicate and absorbances were determined at a wavelength of 450 nm (490 nm for urinary creatinine) using a microplate reader (iMark, Bio-Rad, Hercules, CA, USA). Absorbances of each target were measured against known standard curves. Concentrations were determined using data reduction software (Microplate Manager, version 6, Bio-Rad, Hercules, CA, USA). Intra-assay coefficients of variation of 8.23%, 7.47%, 8.93%, 9.74%, 8.18%, 9.77%, and 8.17% were observed for Nfl, CAF, TNTF, E2, NT, TNF-α, and creatinine, respectively.

### 2.11. Statistical Analysis

Independent group *t*-tests were performed to determine any differences between PRE-M and POST-M women. Effect sizes were calculated using Cohen’s *d* (*d* = 0.2 is considered a “small” effect size, 0.5 represents a “medium” effect size, and 0.8 a “large” effect size) [26] and corrected for a sample size less than [27]. All statistical procedures were performed using SPSS version 23.0 software (SPSS, Chicago, IL, USA) and an alpha level of *p* ≤ 0.05 was adopted throughout.

## 3. Results

### 3.1. Body Composition

The outcomes for body composition for each group are presented in Table 1. Significant differences were observed for TBM (*p* = 0.02, *d* = 1.56), PBF (*p* = 0.01, *d* = 1.76), FM (*p* = 0.01, *d* = 1.74), and VFM (*p* < 0.001, *d* = 3.49) where the values for POST-M women were higher than those for PRE-M women. There were no significant differences between groups for TBW (*p* > 0.05).

### 3.2. Fat-Free Mass and Muscle Quality Scores

The results for FFM and MQ are presented in Table 2. For FFM, the results showed no significant differences for the arms, legs, and arms and legs (appendicular) combined (*p* > 0.05). However, the MQ scores demonstrated significant differences in both the upper (*p* = 0.009, *d* = 1.75) and lower body (*p* = 0.025, *d* = 1.36), thereby indicating PRE-M women to have superior MQ than POST-M women.

### 3.3. Muscular Performance and Strength

The results for absolute and relative strength and muscle endurance are presented in Table 3. For the chest press and leg press, both groups established their 1RM with three attempts at each exercise. For absolute strength, there were no significant differences between groups for the upper and lower body (*p* > 0.05). However, there was a moderate trend towards significance (*p* = 0.07) with a strong effect size (*d* = 0.96) favoring PRE-M women. There were also no significant differences between groups for upper- and lower-body muscle endurance using RTF at 70% 1RM (*p* > 0.05). However, when muscle strength was expressed relative to total body mass, significant differences were noted indicating PRE-M women to be stronger regarding their upper (*p* = 0.016, *d* = 1.82) and lower body (*p* = 0.043, *d* = 1.17) compared to POST-M women.

### 3.4. Muscle Activation

The results of motor unit and muscle activation patterns are presented in Table 4. There were significant differences between PRE-M and POST-M women in all measured variables, indicating POST-M women to be undergoing less motor unit and muscle activation than PRE-M women. Maximum VL activation was observed to be significantly higher in PRE-M women for both 1RM (*p* = 0.012, *d* = 1.65) and for maximum (*p* = 0.014, *d* = 1.71) and mean activation (*p* = 0.032, *d* = 1.39) while performing RTF at 70% 1RM. Similarly, RTF maximum activation was observed in both 1RM (*p* = 0.003, *d* = 2.25) and RTF at 70% 1RM (*p* = 0.042, *d* = 1.38) to be greater in PRE-M women. In addition, the mean RTF muscle activation while performing RTF at 70% 1RM was greater in PRE-M women (*p* = 0.024, *d* = 1.51) when compared to POST-M women.

### 3.5. Serum and Urinary Analyses

The results for serum and urinary protein and hormonal concentrations are presented in Table 5. There was a significant difference between groups in serum E2 (*p* < 0.00, *d* = 25.79), demonstrating PRE-M women to have greater levels of E2 than POST-M women. However, there were also significant differences between groups in serum CAF (*p* < 0.001, *d* = 5.54), TNTF (*p* < 0.001, *d* = 4.36), and TNF-α (*p* < 0.0001, *d* = 4.81), in which the values for POST-M women were greater than those for PRE-M women. Regarding NfL and NT, there were no significant differences between groups (*p* > 0.05); however, NT was greater in POST-M women, with a moderate trend towards significance (*p* = 0.07) and a strong effect size of 1.02.

### 3.6. Dietary Intake

Dietary intake results can be seen in Table 6 and, between groups, there were no significant differences in macronutrient intake for carbohydrate, fat, and total calories (*p* > 0.05). Protein intake for PRE-M women was shown to be significantly greater compared to POST-M women (*p* = 0.011, *d* = 2.53). While the total calorie intake was greater for PRE-M women, there was not a significant difference; however, there was a moderate trend towards significance (*p* = 0.067) and a strong effect size (*d* = 1.37).

## 4. Discussion

Menopause leads to a cascade of physiological changes beginning with the decline in endogenous E2. Normal E2 ranges for menstruating women fluctuate between 30 and 400 pg/mL based on the phases of the uterine cycle; however, the levels are 15 to 40 pg/mL in post-menopausal women [28]. In our present study, in addition to the self-reports of menstrual activity status from participants, the PRE-M and POST-M groups presented with E2 concentrations of 399.41 pg/mL (all PRE-M participants were assessed during the follicular phase of their uterine cycle) and 26.36 pg/mL, respectively, supporting the normal clinically accepted ranges for both groups, and verifying that the POST-M women were indeed post-menopausal. In the present study, the significantly lower levels of E2 in the POST-M group occurred in conjunction with significantly greater levels of CAF, NT, TNTF, and TNF-α. Furthermore, POST-M women were shown to have significantly less motor unit/muscle activation, MQ, and muscle strength.

E2 has the capability of preventing neuronal cell death caused by increased oxidative burden. The antioxidant and neuroprotective effects of E2 are dependent not on its genomic properties as a hormone but rather on the phenolic structure of the A-ring and its basic chemical properties as a hydrophobic phenolic molecule [29]. E2 can protect against free radical generators [30] because it lowers peroxide production. As such, E2 has been shown to up-regulate the transcription rate and increase the enzyme activity of manganese and extracellular isozymes of superoxide dismutase [31], catalase, and glutathione peroxidase [32]. In addition to its antioxidant effects, E2 binds to estrogen receptors (ER) and up-regulates the expression of antioxidant enzymes via intracellular signaling pathways. As a result, E2 inhibits inflammatory cytokine and chemokine expression via an ER-dependent mechanism [33]. It has been shown that ER-α, but not ER-β, is required for the neuroprotective effects of E2, and this is at least partially due to E2 up-regulating the anti-apoptotic bcl-2 mRNA expression, thereby decreasing mitochondrial apoptosis [34]. Thus, through modulation of antioxidant enzyme expression and the activity and inhibition of apoptosis, E2 can prevent neuronal decline by blocking increases in reactive oxygen species production and apoptotic-induced mitochondrial/cellular damage [35].

Along with muscular changes within the muscle fibers, neuromuscular changes are seen in association with advancing age [36]. Agrin, a heparan sulfate proteoglycan, is an important protein whose role maintains the structural integrity of the NMJ through post-synaptic differentiation and acetylcholine receptor (AChR) clustering [37]. The proteolytic target of the trypsin-like serine protease, neurotrypsin, is agrin [13]. There is a homeostatic relationship between the degradation of agrin into CAF by NT and the protection and maintenance of the stability of the NMJ during periods of denervation and reinnervation. Furthermore, NT may be necessary for the reorganization of the NMJ to ensure synaptic plasticity [13]. However, our data suggest that the reduction in endogenous E2 appeared to up-regulate NT activity, as we observed a significantly greater amount of circulating NT (137.13 vs. 405.50 ng/mL) and CAF (1208.40 vs. 3860.20 pg/mL) in POST-M women. Even though we did not observe a significant difference in NT between groups, our results indicated a moderate trend towards significance (*p* = 0.07) and a strong effect size (*d* = 1.02); therefore, with a larger sample size, we are confident a significant difference would have been observed.

Neurotrypsin is stored in presynaptic nerve endings and secreted in an inactive zymogenic form by synaptic activity at the NMJ. After activation, which requires *N*-methyl-*D*-aspartate (NMDA) receptor up-regulation [38], NT cleaves agrin to form CAF [39]. Furthermore, there are data demonstrating the NMDA receptor to be up-regulated by TNF-α [40]. Increased NMDA receptor activity can facilitate the excitotoxic death mechanism within neurons [41]. Furthermore, the cytoplasmic domain of the TNF receptor contains a ‘death domain’ sequence responsible for transducing the NF-кB signaling pathway and apoptosis-related caspases [42]. In the present study, the oxidative stress and pro-inflammatory release of TNF-α conceivably caused by the reduced endogenous E2 observed in POST-M women may likely have been associated with the increased concentration of NT and CAF we observed to occur in POST-M women.

E2 is an inhibitor of the pro-inflammatory cytokine, TNF-α [43]. However, the decline in E2 exposes myofibers to oxidative stress and chronic inflammation, which instigates proteolytic activity, thereby disrupting the normal protein turnover process [6]. Increased levels of TNF-α also inhibit the expression and activity of anabolic hormones such as growth hormone and insulin-like growth factor-1 (IGF-1) [44]. Mechanistically, TNF-α receptor activation is known to up-regulate NFкB by signaling through the IкB kinase (IKK) complex pathway. NF-кB activation can also interfere with myogenic differentiation typically required for the regeneration of atrophied skeletal muscle [45] occurring during sarcopenia. NF-кB is a transcription factor which plays a role in the transactivation of various genes in skeletal muscle involved in the ATP-dependent ubiquitin proteolytic pathway [46], which is a major pathway for the proteolytic degradation of intact sarcomeric proteins, such as titin. However, since we did not obtain muscle biopsies, we were unable to assess NF-кB activation status; therefore, we can only speculate as to its role in mediating the processes associated with titin degradation.

Sarcopenia is a muscular disease, and TNTF has been considered a clinical biomarker for the early indication of muscle wasting leading up to sarcopenia [22]. We observed TNTF concentrations to be significantly greater in POST-M compared to PRE-M women (20.69 vs. 11.10 ng/mL, respectively). Titin degradation is relevant in that it is a structural protein aiding in the maintenance and integrity of the sarcomere. It is conceivable that our results indicate that the increased proteolytic activity of this protein played a subsequent mechanistic role in the decreased MQ we observed in POST-M women. Ultimately, our study suggests that the low levels of E2 we observed in early to intermediate post-menopausal women were likely associated with the increases occurring in NT, CAF, and TNF-α, a cascade that was conceivably leading to the proteolytic degradation of titin and subsequent decreases in motor unit recruitment and activation and MQ. Functionally, it has been shown that these are all characteristics related to sarcopenia leading into, and during, late post-menopause [47].

Aging is associated with a reduction in the number of functioning motor units, which directly contributes to muscle weakness [48]. As the NMJ ages and CAF levels increase, AChR clusters break into fragments that become poorly innervated [37]. Despite no significant differences between groups relative to NfL, a protein that provides evidence of deterioration at the axonal level, our data imply that NfL does not play a direct role in NMJ integrity and motor unit activation and MQ. Furthermore, regarding NMJ formation, agrin signaling is required for NMJ stability. The degradation of agrin to CAF by NT results in the deterioration of the NMJ, which contributes to muscle atrophy and weakness [12]. Post-menopausal women have been shown to have elevated levels of CAF [16,49]. In the present study, since we observed POST-M women to have less E2, but greater levels of NT and CAF, than PRE-M women, it is likely that the apparent E2-induced increase in CAF may have detrimentally impacted NMJ integrity, thereby playing a role in the significantly decreased motor unit/muscle activation and MQ we also observed.

One of the most commonly cited neuromuscular changes associated with aging is a reduction in the functioning of motor units [48]. There are age-related changes in motor unit morphology and properties resulting in decreased AChR clustering and NMJ degradation that lead to impaired motor performance and include reduced maximal strength and power, slower contractile velocity, and increased fatigability. Primary factors underlying these changes and instability of the NMJ are known to involve greater mitochondrial dysfunction of pre- and post-synaptic terminals, oxidative stress, and inflammation which lead to aging-related neurodegeneration [50]. We observed motor unit recruitment/muscle activation to be lower in POST-M women in the presence of elevated CAF, NT, TNTF, and TNF-α, all which are conceivably linked to reduced E2. In the present study, considering motor unit activation, the maximum amount of activation was significantly greater for PRE-M women in both the vastus lateralis and rectus femoris muscles during the 1RM leg press attempts. We observed the same result favoring PRE-M women for the mean amount of activation occurring in the vastus lateralis and rectus femoris muscles throughout the course of the muscle endurance testing (repetitions to failure at 70% 1RM). Interestingly, PRE-M women had more detectable activation than POST-M women in the leg press 1RM and leg press RTF for both the VL and RF. These findings support the known neuromuscular decline associated with advancing age [34].

Even though PRE-M women demonstrated significantly greater upper- and lower-body muscle relative strength, both groups had similar appendicular fat-free mass (17.05 kg in PRE-M women vs. 17.86 kg in POST-M women). However, when we further expressed muscle mass and strength in terms of MQ, PRE-M women displayed significantly greater MQ for the upper and lower body when compared to POST-M women. The relative strength and endurance for the upper body and the lower body were significantly greater for PRE-M compared to POST-M women.

Several factors may contribute to low muscle mass and MQ. Risk factors for low muscle mass include age, inadequate protein intake, physical inactivity [51], neurodegenerative disease, hormone deficiency and systemic inflammation [52]. During aging, an adequate protein intake is important for maintaining muscle mass, as insufficient protein intake increases the rate of muscle mass loss [53], strength [54] and performance [55]. We observed PRE-M women to be ingesting 1.47 g/kg/day of protein, whereas POST-M women ingested 0.81 g/kg/day. As the recommended dietary allowance (RDA) recommends a daily protein intake of 0.8–1.0 g/kg for the general population, this suggests our POST-M participants to be ingesting adequate protein. A protein intake exceeding the current RDA has been proposed as a strategy to preserve muscle mass and physical function in advancing age [56,57,58]. Furthermore, a recent review suggested that older adults need to ingest 1.0–1.3 g/kg/day of protein to sustain muscle mass and physical functionality [53,59]. Relatedly, regarding dietary intake, both groups were ingesting equivalent amounts of carbohydrate and fat; however, POST-M women ingested fewer daily calories in total than PRE-M women. This difference in daily caloric intake was not significantly different between groups but had a moderate trend towards significance (*p* = 0.067) with a strong effect size. (*d* = 1.37). Even though the dietary intakes were only recorded for three days, this indicates that not only did POST-M women consume fewer calories during that time, they also consumed less protein. Furthermore, if this information is indicative of the participants’ typical dietary intake, since consuming adequate amounts of calories and protein plays a crucial role in energy availability and facilitating muscle protein synthesis, this may have played a role in the reduced MQ we observed for POST-M women. We realize that our small sample size may not represent an accurate reflection of protein intake in post-menopausal women. However, a recent study surveyed 5732 older females (average age 79 years) and found that only 56% of participants met the RDA for protein of 0.8 g/kg/day [60]. The results of this study provide some justification that the low protein intakes we observed in our small sample of post-menopausal women may in fact be an accurate representation of what has been shown to exist in a much larger sample of older women.

Along with a reduced MQ, we also observed POST-M women to have significantly greater amounts of FM and VFM. This was not an unexpected result because it has been widely documented that as individuals age, the body composition changes, even in the absence of changes in body weight. Studies have shown that fat mass increases and muscle mass decreases with age [61]. Menopause is associated with significant changes in body composition and the accumulation of peri-abdominal or visceral fat and a subsequent reduction in FFM that is also associated with changes in resting energy expenditure and spontaneous activity. A reduction in E2 levels seems to be an important trigger for these changes [62]. For example, ovarian suppression using a GnRH antagonist has shown that the correction of the ensuing E2 deficiency preserved fat-free mass and resting energy expenditure and prevented the increase in abdominal subcutaneous and visceral adipose tissue seen with the unopposed use of the GnRH antagonist. The women receiving the E2 were also more physically active than those who were E2-deficient [63].

The limitations of this study include the small sample size. However, we calculated effect sizes to quantify the strength of the data. All data resulting in significant differences had strong effect sizes. Also, in some instances such as NT, where significant differences were not detected, there was a moderate trend toward significance associated with a strong effect size. Therefore, we are confident in our results but also realized that a larger sample size would have strengthened the interpretability of the study outcomes. Additionally, surface EMG is often considered to have limitations in quantifying activation due to muscle crosstalk based on electrode placement and signal sensitivity due to adipose tissue thickness. We reduced the chance of crosstalk by ensuring that electrodes were placed at a substantial distance from one another. Also, there was no significant difference between groups for leg fat mass. Therefore, we feel confident that appropriate steps were taken to adequately reduce the limitations of the EMG that allowed us to collect valid data. Unfortunately, our EMG system was not able to perform decomposition assessment; therefore, we were unable to estimate motor unit activation speed and determine muscle fiber type.

## 5. Conclusions

For biological women, muscle loss is a predictable outcome during the advancing stages of menopause. As such, the risk of sarcopenia increases with progressive decreases in circulating E2. This is noteworthy since many women will live almost half their lives with reduced E2 concentrations [64]. In the cohort of early to intermediate cohort post-menopausal women in our study, we conclude that reductions in E2, protein intake, and antioxidant effects most likely up-regulated TNF-α levels, which subsequently (1) increased the activity of NT to degrade agrin to CAF and (2) up-regulated the activity of NF-кB to instigate muscle proteolysis, thereby resulting in increased TNTF. We also conclude that the culminating effects of this inflammatory and proteolytic cascade conceivably had a detrimental impact on NMJ integrity, motor unit activation, and MQ compared to the pre-menopausal women, who displayed normal levels of E2.

## Figures and Tables

**Table 1:** Descriptive data for body composition variables.

Body Composition Variables	Group	Mean ± SD	*p*-Value	Cohen’s *d*
Total Body Mass (kg)	PRE-M	60.56 ± 11.60	0.02 *	1.56
	POST-M	77.13 ± 9.31		
Total Body Water (L)	PRE-M	30.31 ± 3.96	0.14	0.71
	POST-M	32.66 ± 2.38		
Percent Body Fat (%)	PRE-M	30.52 ± 8.42	0.01 *	1.76
	POST-M	41.84 ± 3.57		
Fat Mass (kg)	PRE-M	19.07 ± 8.41	0.01 *	1.74
	POST-M	32.26 ± 6.46		
Visceral Fat Mass (kg)	PRE-M	0.26 ± 0.21	<0.001 *	3.49
	POST-M	0.92 ± 0.24		

* Indicates that the value for POST-M women was significantly greater than that for PRE-M women, *p* ≤ 0.05.

**Table 2:** Descriptive data for fat-free mass and muscle quality variables.

Muscle Composition Variables	Group	Mean ± SD	*p*-Value	Cohen’s *d*
Arms Fat-Free Mass (kg)	PRE-M	3.98 ± 0.7	0.13	0.74
	POST-M	4.33 ± 0.3		
Legs Fat-Free Mass (kg)	PRE-M	13.28 ± 1.5	0.33	0.28
	POST-M	13.61 ± 1.6		
Trunk Fat-Free Mass (kg)	PRE-M	19.13 ± 2.25	0.069	1.25
	POST-M	21.57 ± 1.5		
Appendicular Fat-Free Mass (kg)	PRE-M	17.1 ± 2.0	0.25	0.43
	POST-M	17.9 ± 1.7		
Upper Body Muscle Quality (RS/FFM)	PRE-M	0.05 ± 0.01	0.009 *	1.75
	POST-M	0.02 ± 0.02		
Lower Body Muscle Quality (RS/FFM)	PRE-M	0.13 ± 0.02	0.025 *	1.36
	POST-M	0.09 ± 0.03		

RS = relative strength; * indicates that the value for POST-M women was significantly less than that for PRE-M women, *p* ≤ 0.05.

**Table 3:** Descriptive data for muscle strength and endurance variables.

Muscular Strength Variables	Group	Mean ± SD	*p*-Value	Cohen’s *d*
Upper-Body 1RM (kg)	PRE-M	36.65 ± 6.31	0.07	0.96
	POST-M	29.02 ± 9.67		
Lower-Body 1RM (kg)	PRE-M	100.15 ± 17.11	0.37	0.21
	POST-M	94.33 ± 36.58		
Upper-Body Relative Strength (kg/kg)	PRE-M	0.58 ± 0.07	0.016 *	1.82
	POST-M	0.38 ± 0.14		
Lower-Body Relative Strength (kg/kg)	PRE-M	1.67 ± 0.24	0.043 *	1.17
	POST-M	1.23 ± 0.51		
Upper-Body Endurance (RTF at 70% 1RM)	PRE-M	14.5 ± 3.4	0.38	0.18
	POST-M	13.6 ± 5.9		
Lower-Body Endurance (RTF at 70% 1RM)	PRE-M	24.3 ± 8.8	0.37	0.22
	POST-M	22.8 ± 2.9		

RTF = repetitions to failure; * indicates that the value POST-M women was significantly less than that for PRE-M women, *p* ≤ 0.05.

**Table 4:** Descriptive data for motor unit and muscle activation variables.

Muscle Activation	Group	Mean ± SD	*p*-Value	Cohen’s *d*
Vastus Lateralis 1RM Max (mV)	PRE-M	0.24 ± 0.07	0.012 *	1.65
	POST-M	0.12 ± 0.08		
Rectus Femoris 1RM Max (mV)	PRE-M	0.23 ± 0.09	0.003 *	2.25
	POST-M	0.08 ± 0.04		
Vastus Lateralis RTF Max (mV)	PRE-M	0.23 ± 0.09	0.014 *	1.71
	POST-M	0.10 ± 0.04		
Rectus Femoris RTF Max (mV)	PRE-M	0.23 ± 0.13	0.042 *	1.38
	POST-M	0.10 ± 0.02		
Vastus Lateralis RTF Mean (mV)	PRE-M	0.11 ± 0.04	0.032 *	1.39
	POST-M	0.06 ± 0.03		
Rectus Femoris RTF Mean (mV)	PRE-M	0.09 ± 0.05	0.024 *	1.51
	POST-M	0.03 ± 0.01		

RTF = repetitions to failure; RTF Max = maximum EMG activity during the course of RTF; RTF Mean = mean EMG activation during the course of RTF; * indicates that the value for POST-M women was significantly less than that for PRE-M women, *p* ≤ 0.05.

**Table 5:** Descriptive data for biochemical variables.

Proteins and Hormones	Group	Mean ± SD	*p*-Value	Cohen’s *d*
*C*-Terminal Agrin Fragment (pg/mL)	PRE-M	1208.40 ± 370.78	<0.001 ^†^	5.54
	POST-M	3860.20 ± 566.43		
Titin *N*-Terminal Fragment (ng/mL)	PRE-M	11.1 ± 1.24	<0.001 ^†^	4.36
	POST-M	20.69 ± 2.84		
Neurofilament Light Chain (ng/mL)	PRE-M	100.88 ± 6.75	0.486	0.02
	POST-M	100.99 ± 2.89		
Estradiol (pg/mL)	PRE-M	399.41 ± 20.31	<0.001 *	25.79
	POST-M	26.36 ± 2.45		
Neurotrypsin (ng/mL)	PRE-M	253.02 ± 44.79	0.07	1.02
	POST-M	329.81 ± 12.59		
Tumor Necrosis Factor-α (pg/mL)	PRE-M	137.13 ± 34.80	<0.001 ^†^	4.81
	POST-M	405.50 ± 70.72		

*C*-terminal agrin fragment = CAF; titin *N*-terminal fragment = TNTF, expressed relative to urinary creatinine; neurofilament light chain = NfL; estradiol = E2; neurotrypsin = NT; tumor necrosis factor-α (TNF-α); * indicates that the value for POST-M women is significantly less than that for PRE-M women; ^†^ indicates that the value for POST-M women was significantly greater than that for PRE-M women, *p* ≤ 0.05.

**Table 6:** Descriptive data for dietary intake variables.

Dietary Intake	Group	Mean ± SD	*p*-Value	Cohen’s *d*
Protein (g/kg)	PRE-M	1.47 ± 0.27	0.011 *	2.53
	POST-M	0.81 ± 0.23		
Carbohydrate (g/kg)	PRE-M	4.28 ± 1.23	0.48	0.64
	POST-M	2.91 ± 2.79		
Fat (g/kg)	PRE-M	1.02 ± 0.34	0.37	0.82
	POST-M	0.79 ± 0.22		
Total Calories (kcal/kg)	PRE-M	32.07 ± 8.23	0.067	1.37
	POST-M	19.58 ± 9.68		

* indicates that the value for POST-M women was significantly less than that for PRE-M women, *p* ≤ 0.05.

## Data Availability

Data are contained within the article.
